# Supplementary material for: Habitat sharing and interspecies interactions in caves used by bats in the Republic of Congo
Source: PeerJ. 2025 Jan 9;13:e18145. doi: 10.7717/peerj.18145 (PMC11725272; doi:10.7717/peerj.18145)

Appendix S1: General diagram of the configuration of the Mont Belo cave, the location of the camera trap and the bat roosting sites. Silhouette image source credit: Microsoft. Photograph image source credit: ML.

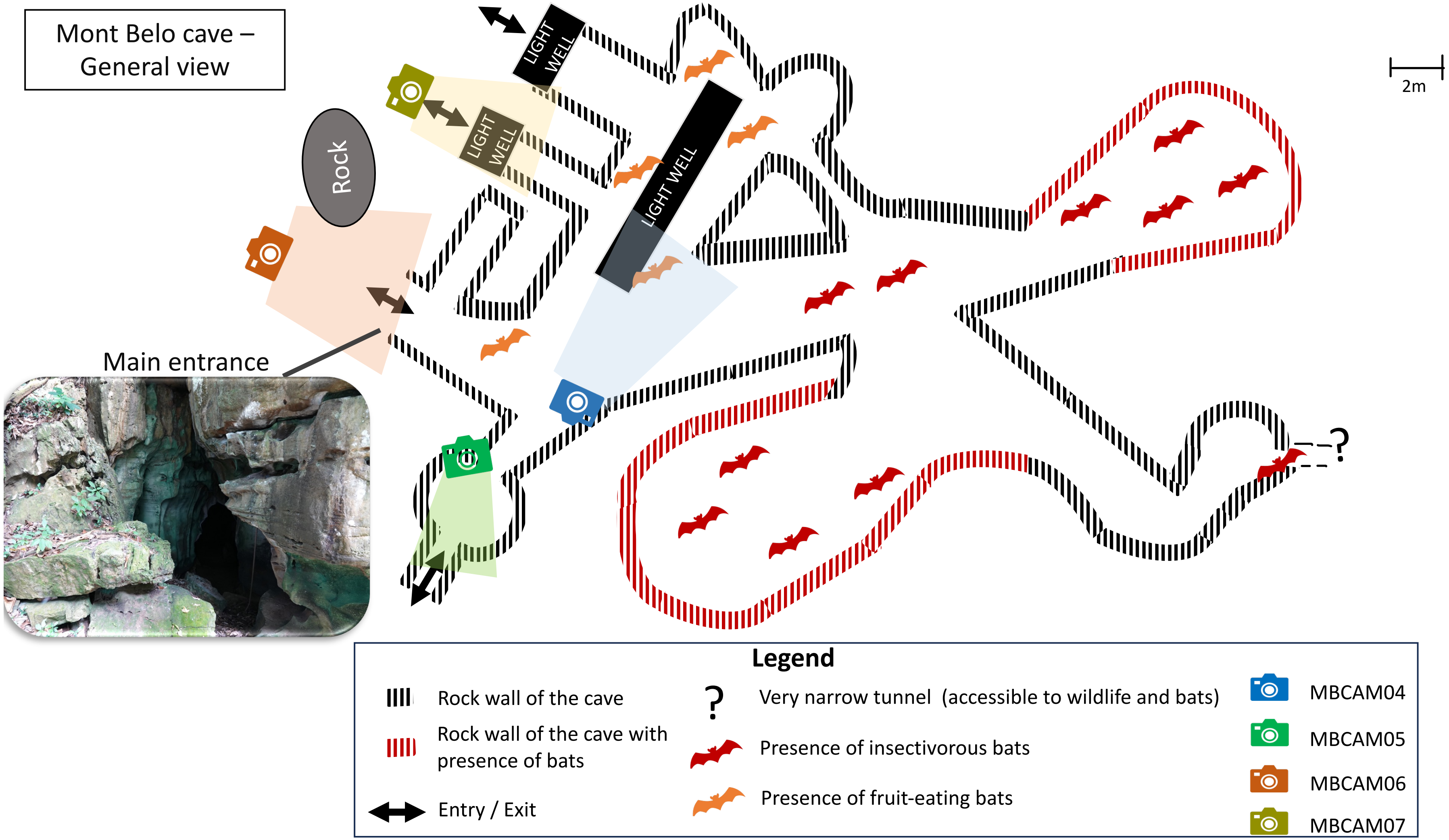

Appendix S2: General diagram of the configuration of the Boundou cave, the location of the camera trap and the bat roosting sites. Silhouette image source credit: Microsoft. Photograph image source credit: ML.

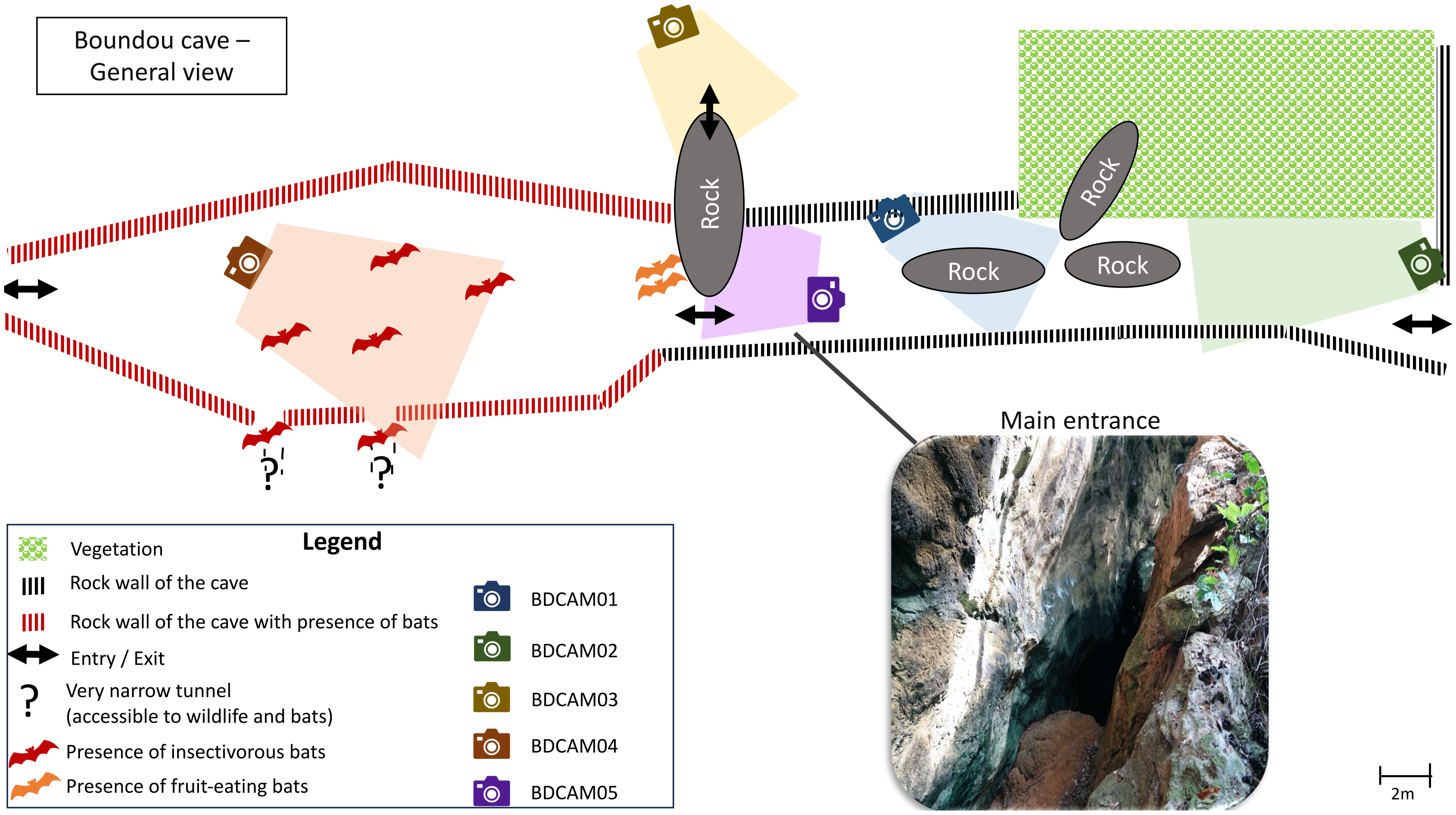

Appendix S3: Details of rodent capture sessions in the two caves in this study.

During these capture sessions, we used two types of trap to capture rodents (Tomahawk and Havahart). In total, 16 traps were set in Mont Belo (nine Tomahawk and seven Havahart), due to the high detection of rodents in this cave, and six traps in the Boundou cave (three Tomahawk and three Havahart).

The traps were set each time on two consecutive nights by placing several types of bait (groundnuts, sardines, corn, etc.) inside the traps. The traps were baited in the early morning each time and checked the next morning.

We caught two small rodents in the Boundou cave and none in the Boundou cave. Preliminary identification identifies this species as *Prionomys batesi*, although genetic analysis is underway to confirm this information.

Appendix S4*:* Raw data of detection

https://dataverse.cirad.fr/privateurl.xhtml?token=a3f476f6-1bfa-4a0b-b88b-46f90909b262

Appendix S5: Number of days the camera traps were in operation during the study. A total of nine cameras for all sites, with five for Boundou cave (including one inside the cave) and four for Mont Belo cave (including 2 inside the cave).

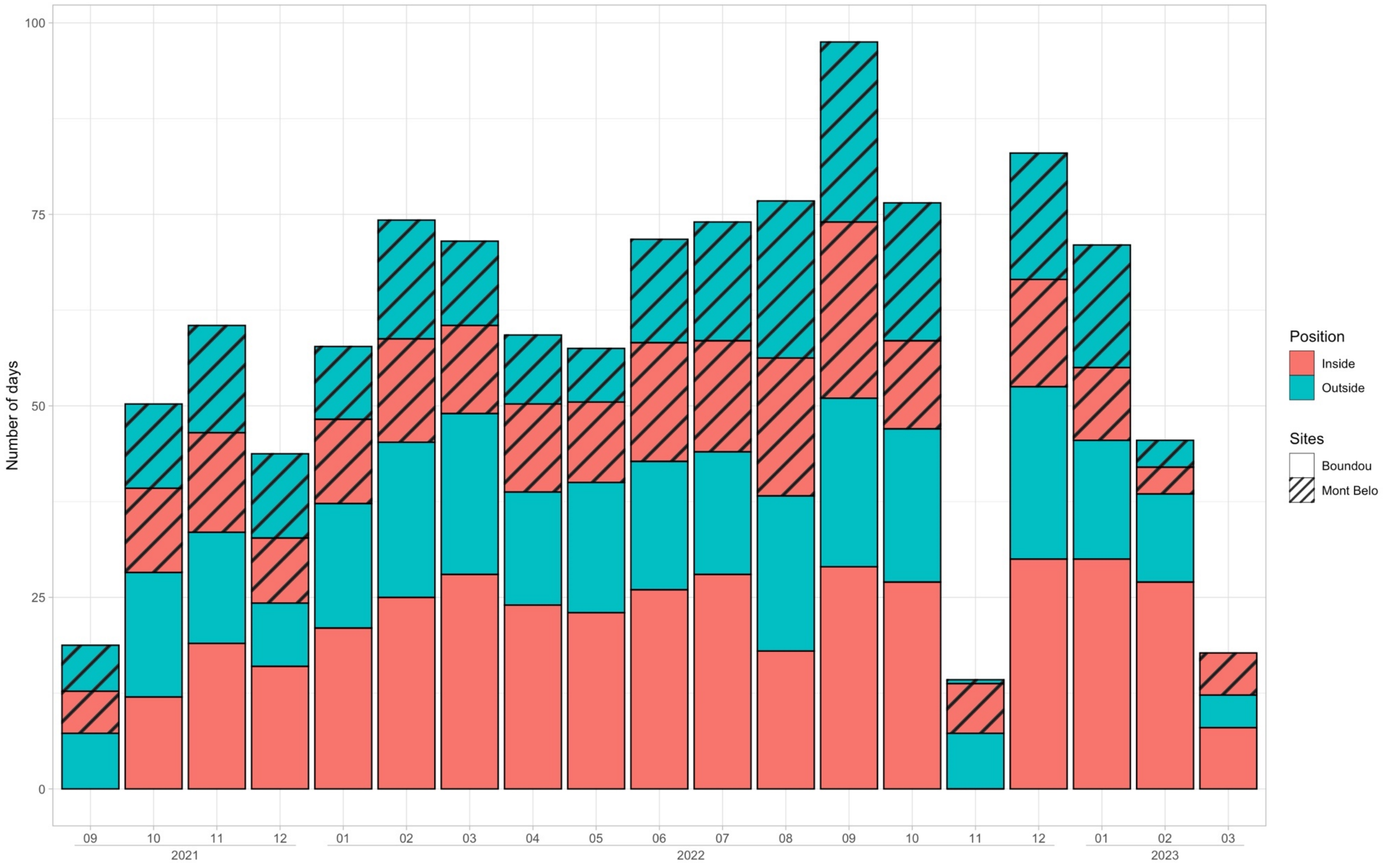

Appendix S6: Number of vertebrate species per camera days multiplied by 100 according to study sites (Boundou and Mont Belo cave) and position of camera (inside or outside). This figure shows all the detections.

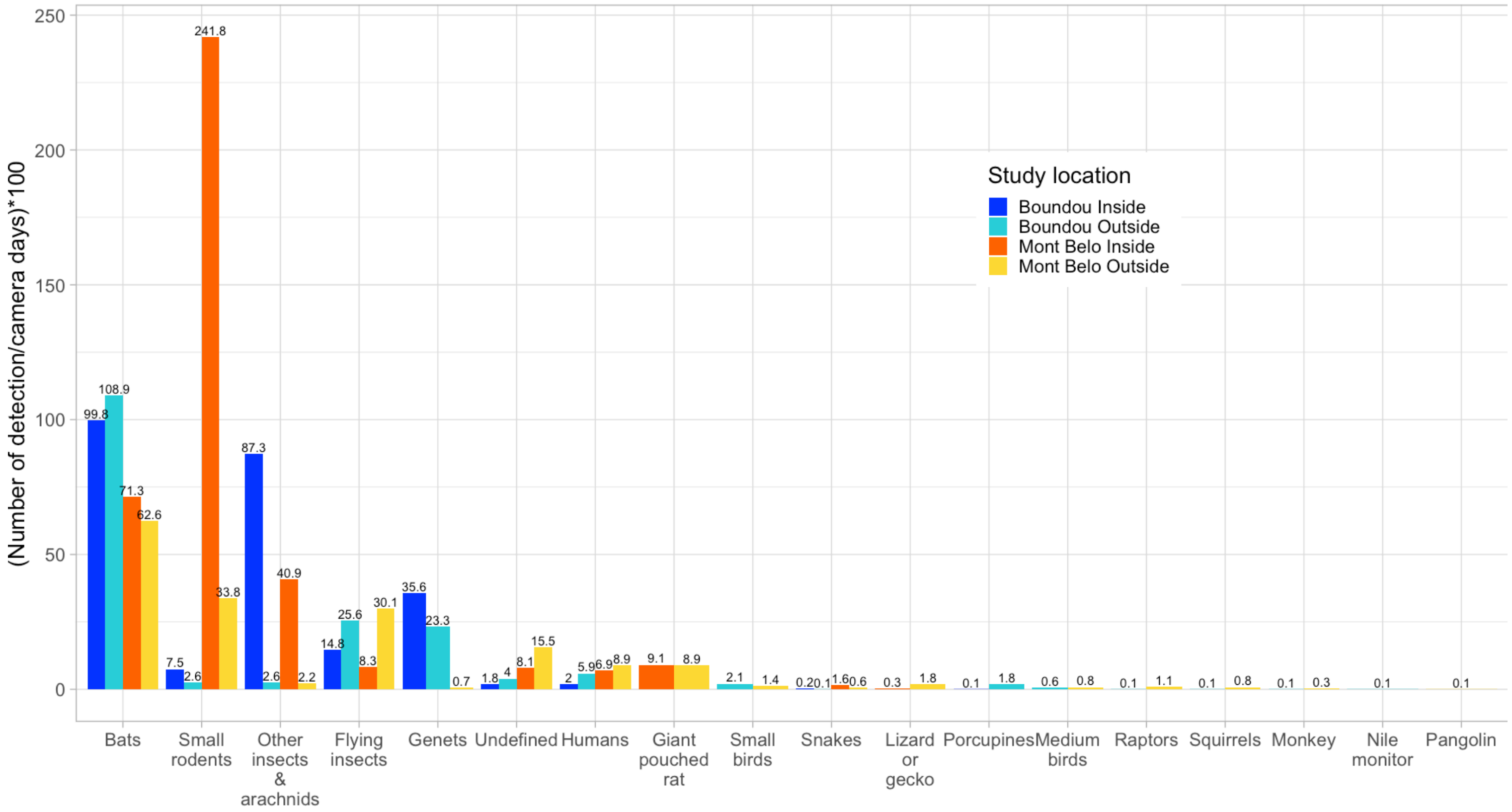

Appendix S7: List of all detected species and scientific name if available (N = 11,581 detections for a total of 11,767 animals)

Common names	Scientific Name	Group	Study site and posi-	Number of detection	Number of animals
African harrier hawk	Polyboroides typus	Raptors	Boundou Outside	1	1
African wood owl	Strix woodfordii	Raptors	Mont Belo Outside	10	10
Ants	NA	Other insects	Mont Belo Outside	1	1
Bats	NA	Bats	Boundou Inside	1562	1562
Bats	NA	Bats	Boundou Outside	1705	1705
Bats	NA	Bats	Mont Belo Inside	626	626
Bats	NA	Bats	Mont Belo Outside	550	550
Large rodents	Cricetomys emini	Giant pouched rat	Mont Belo Inside	79	79
Large rodents	Cricetomys emini	Giant pouched rat	Mont Belo Outside	78	78
Large rodents	NA	Large rodents	Mont Belo Inside	1	1
Black-bellied seedcracker	Pyrenestes ostrinus	Small birds	Mont Belo Outside	1	1
Butterflies	NA	Flying insects	Boundou Outside	10	10
Butterflies	NA	Flying insects	Mont Belo Outside	13	13
Cave beetles	NA	Other insects	Boundou Inside	1334	1334
Cave beetles	NA	Other insects	Boundou Outside	15	15
Cave beetles	NA	Other insects	Mont Belo Inside	79	79
Genets	Genetta maculata	Genets	Boundou Inside	557	561
Genets	Genetta maculata	Genets	Boundou Outside	364	377
Genets	Genetta servalina	Genets	Mont Belo Outside	6	6
Collared sunbird	Hedydipna collaris	Small birds	Mont Belo Outside	2	2
Crickets	NA	Other insects	Boundou Inside	15	15
Crickets	NA	Other insects	Boundou Outside	10	10
Crickets	NA	Other insects	Mont Belo Inside	253	253
Crickets	NA	Other insects	Mont Belo Outside	4	4
Dove and pigeon	NA	Medium birds	Boundou Outside	10	14
Dove and pigeon	NA	Medium birds	Mont Belo Outside	2	2
Flies	NA	Flying insects	Boundou Inside	232	232
Flies	NA	Flying insects	Boundou Outside	388	388
Flies	NA	Flying insects	Mont Belo Inside	73	73
Flies	NA	Flying insects	Mont Belo Outside	251	251
Giant pangolin	Smutsia gigantea	Pangolin	Mont Belo Outside	1	1
Humans	NA	Humans	Boundou Inside	31	40
Humans	NA	Humans	Boundou Outside	93	98
Humans	NA	Humans	Mont Belo Inside	61	80
Humans	NA	Humans	Mont Belo Outside	78	114

Common names	Scientific Name	Group	Study site and posi-	Number of detection	Number of animals
Icterine greenbul	Phyllastrephus icterinus	Medium birds	Mont Belo Outside	1	1
Lesser striped swallow	Cercropsis abyssinica	Small birds	Mont Belo Outside	8	8
Lizard or gecko	NA	Lizard or gecko	Mont Belo Inside	3	3
Lizard or gecko	NA	Lizard or gecko	Mont Belo Outside	16	16
Monkey	Chlorocebus cynosuros	Monkey	Boundou Outside	1	1
Monkey	Chlorocebus cynosuros	Monkey	Mont Belo Outside	3	3
NI	NA	Not Identified	Boundou Inside	28	29
NI	NA	Not Identified	Boundou Outside	62	71
NI	NA	Not Identified	Mont Belo Inside	71	78
NI	NA	Not Identified	Mont Belo Outside	136	152
Nile monitor	Varanus niloticus	Nile monitor	Boundou Outside	2	2
Palm thrushes	NA	Medium birds	Mont Belo Outside	2	2
Passerine birds	Malimbus sp	Small birds	Boundou Outside	1	1
Pied crow	Corvus albus	Medium birds	Mont Belo Outside	1	1
Porcupines	Atherurus africanus	Porcupines	Boundou Inside	2	2
Porcupines	Atherurus africanus	Porcupines	Boundou Outside	28	29
Scaly spurfowl	Pternistis squamatus	Medium birds	Mont Belo Outside	1	1
Small rodents	NA	Small rodents	Boundou Inside	117	119
Small rodents	NA	Small rodents	Boundou Outside	39	39
Small rodents	NA	Small rodents	Mont Belo Inside	2123	2170
Small rodents	NA	Small rodents	Mont Belo Outside	297	310
Snake	NA	Snakes	Boundou Inside	3	3
Snake	NA	Snakes	Boundou Outside	1	1
Snake	NA	Snakes	Mont Belo Inside	14	14
Snake	NA	Snakes	Mont Belo Outside	5	5
Snowy-crowned robin-chat	Cossyhpa niveicapilla	Small birds	Boundou Outside	32	32
Snowy-crowned robin-chat	Cossyhpa niveicapilla	Small birds	Mont Belo Outside	1	1
Spider	NA	Other insects	Boundou Inside	18	18
Spider	NA	Other insects	Boundou Outside	15	15
Spider	NA	Other insects	Mont Belo Inside	27	27
Spider	NA	Other insects	Mont Belo Outside	14	14
Squirrels	NA	Squirrels	Boundou Outside	1	1
Squirrels	NA	Squirrels	Mont Belo Outside	7	7
Typical striped grass mouse	Lemniscomys striatus	Small rodents	Boundou Outside	2	2
Wasp	NA	Flying insects	Boundou Outside	3	3

30

Appendix S8*:* Facebook of species with camera traps taken during the nineteen months of study. (A) *Genetta servalina*, (B) *Genetta maculata*, (C) *Atherurus africanus*, (D) *Chlorocebus cynosuros*, (E) *small rodents*, (F) *Cricetomys emini*, (G) *Strix woodfordii*, (H) *Cossyhpa niveicapilla* and (I) *Varanus niloticus*. Photograph image source credit: ML.

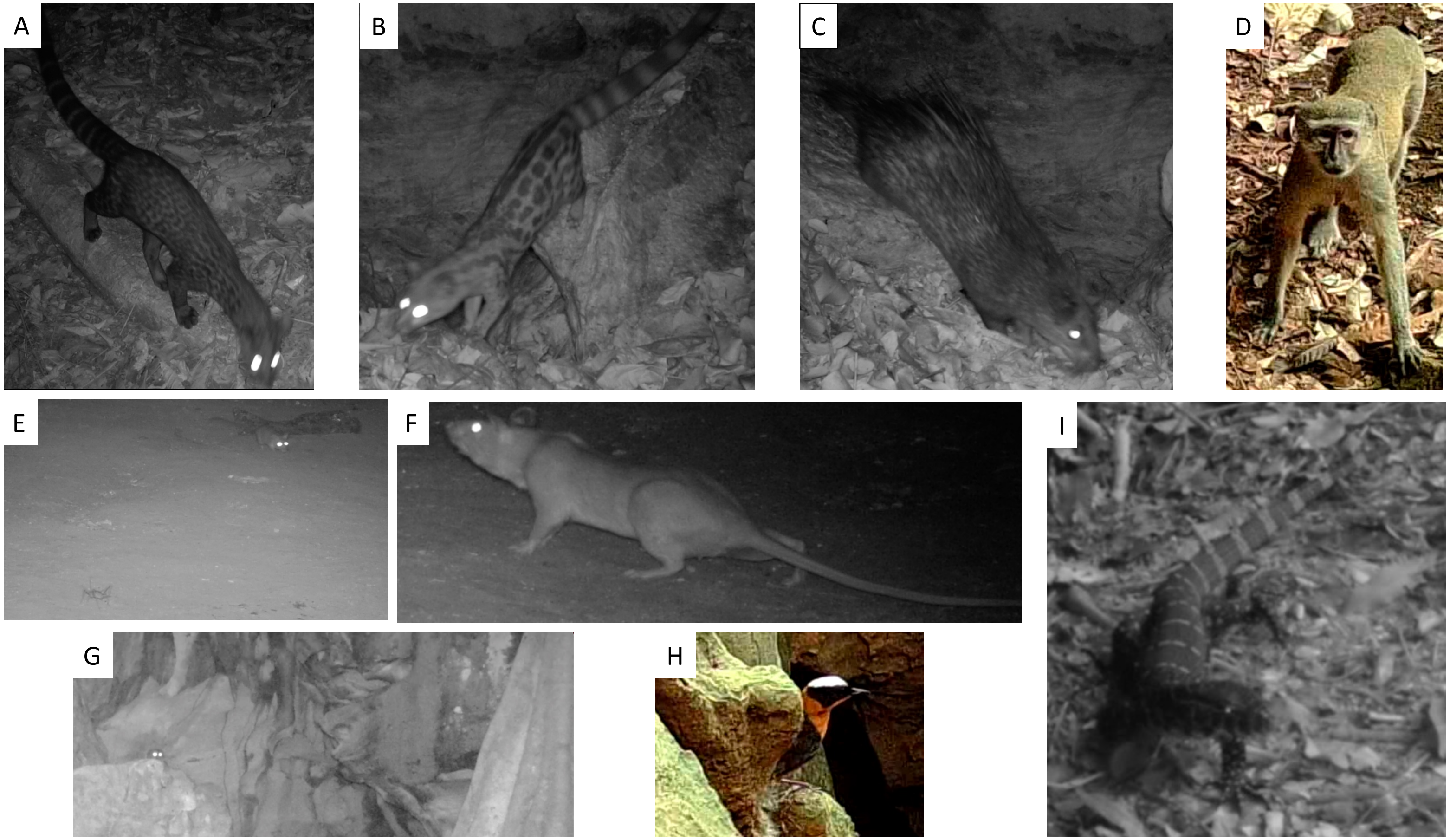

Appendix S9*:* Picture of one individual of Giant pouched rat (*Cricetomys emini*) with swollen jowls. Photograph image source credit: ML.

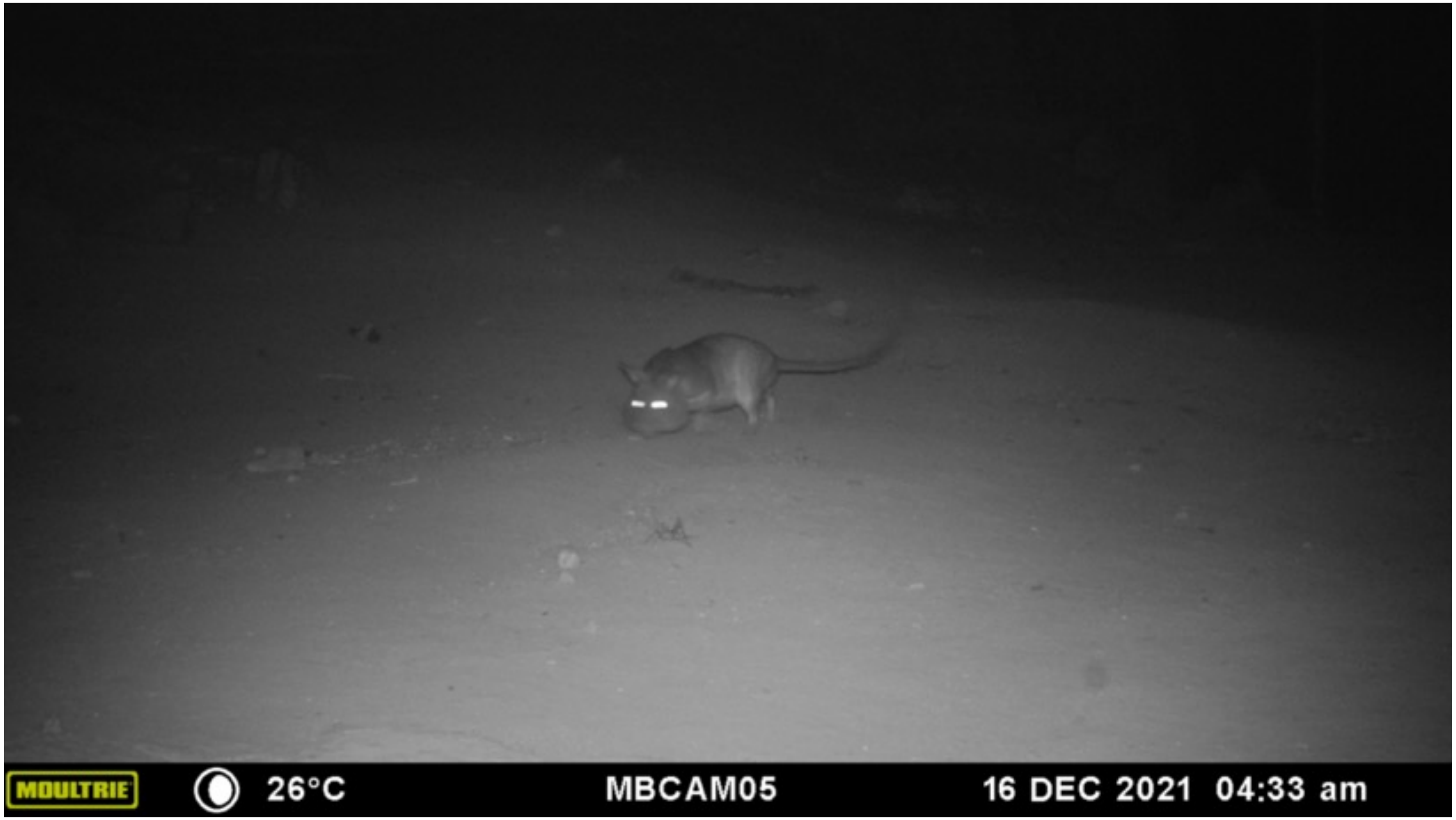

Appendix *S*10: Daily activity pattern of species according to activities outside or inside Mont Belo or Boundou cave. (A, B) overlap activities inside and outside Mont Belo cave for small rodents vs bats, (C, D) overlap activities inside and outside Boundou cave for genets vs bats, (E, F) overlap activities inside and outside Boundou cave for flying insects vs bats, and (G, H) overlap activities inside and outside Mont belo cave for other insects & arachnids vs bats.

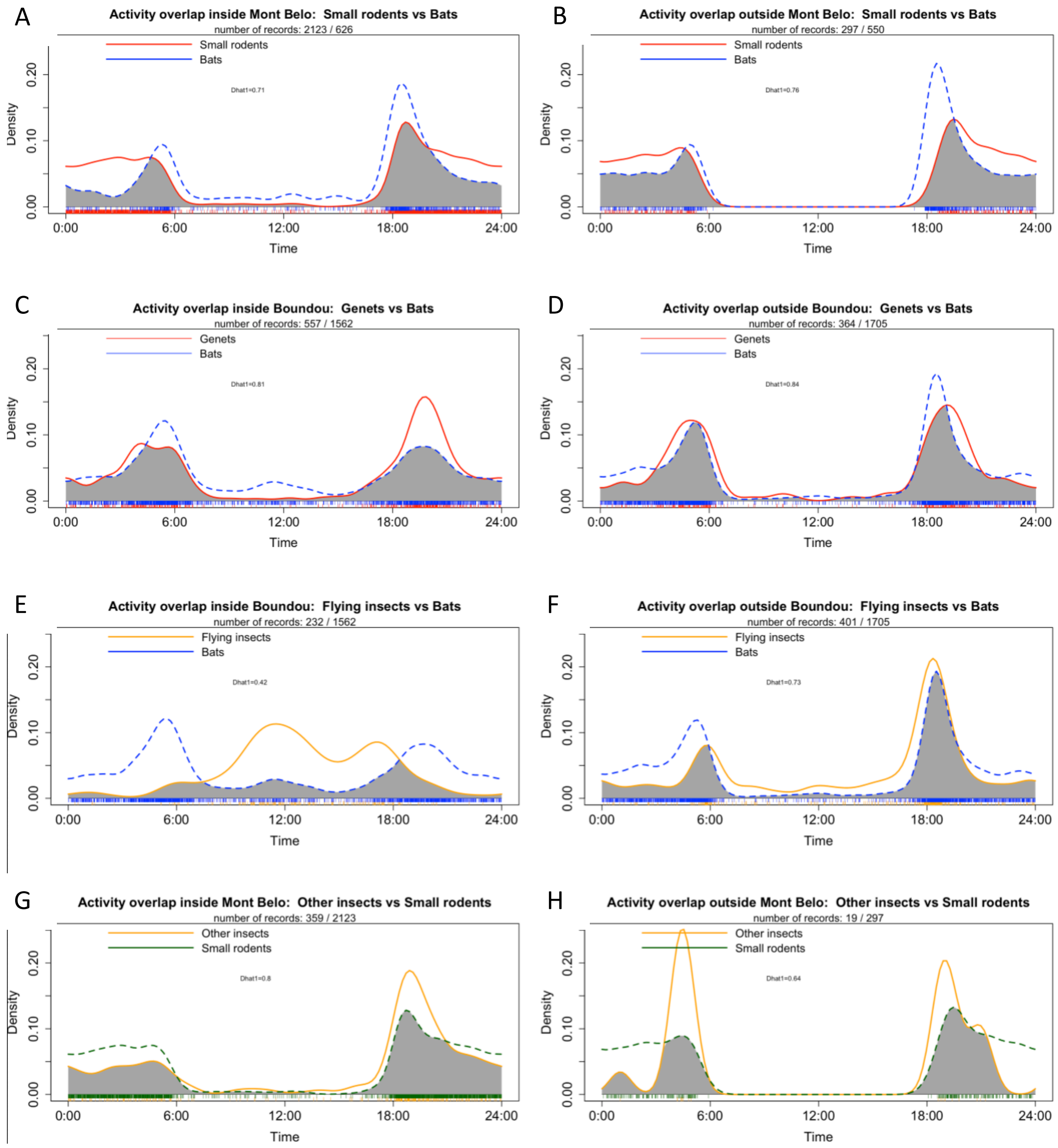

Appendix *S*11*:* Daily activity pattern of species according to activities outside or inside Mont Belo cave. (A, B) overlap activities outside and inside Mont Belo cave for giant pouched rat vs bats (C, D) overlap activities outside and inside Mont Belo cave for flying insects vs small rodents and.

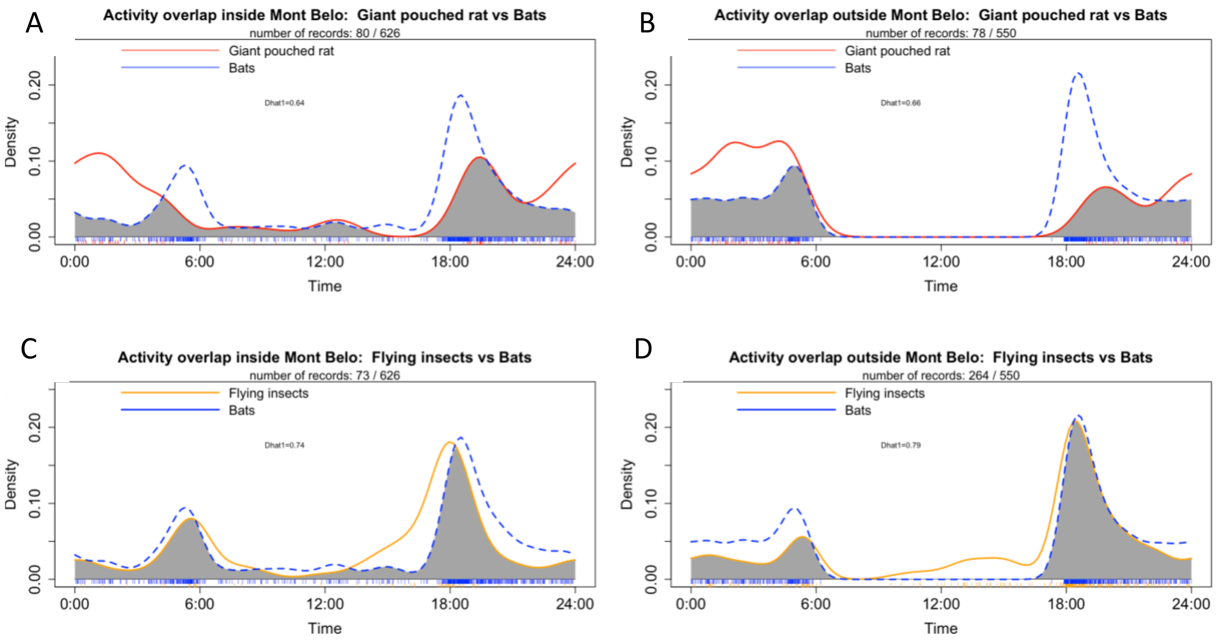

Supplement: Supplemental Information 1 [file peerj-13-18145-s001.pdf]
